# A novel association between relaxin receptor polymorphism and hematopoietic stem cell yield after mobilization

**DOI:** 10.1371/journal.pone.0179986

**Published:** 2017-06-30

**Authors:** Saeam Shin, Juwon Kim, Soo-Zin Kim-Wanner, Halvard Bönig, Sung Ran Cho, Sinyoung Kim, Jong Rak Choi, Kyung-A Lee

**Affiliations:** 1 Department of Laboratory Medicine, Yonsei University College of Medicine, Seoul, Korea; 2 Department of Laboratory Medicine, Hallym University College of Medicine, Kangnam Sacred Heart Hospital, Seoul, Korea; 3 Department of Laboratory Medicine, Yonsei University Wonju College of Medicine, Wonju, Korea; 4 German Red Cross Blood Service BaWüHe, Frankfurt, Germany; 5 Institute for Transfusion Medicine and Immune Hematology of the Johann-Wolfgang-Goethe Medical University, Frankfurt, Germany; 6 Department of Medicine/Hematology, University of Washington, Seattle, Washington, United States of America; 7 Department of Laboratory Medicine, Ajou University School of Medicine, Suwon, Korea; St. Vincent's Institute, AUSTRALIA

## Abstract

Mobilization of hematopoietic stem cells (HSCs) from the bone marrow to the peripheral blood is a complex mechanism that involves adhesive and chemotactic interactions of HSCs as well as their bone marrow microenvironment. In addition to a number of non-genetic factors, genetic susceptibilities also contribute to the mobilization outcome. Identification of genetic factors associated with HSC yield is important to better understand the mechanism behind HSC mobilization. In the present study, we enrolled 148 Korean participants (56 healthy donors and 92 patients) undergoing HSC mobilization for allogeneic or autologous HSC transplantation. Among a total of 53 polymorphisms in 33 candidate genes, one polymorphism (rs11264422) in relaxin/insulin-like family peptide receptor 4 (*RXFP4*) gene was significantly associated with a higher HSC yield after mobilization in Koreans. However, in a set of 101 Europeans, no association was found between circulating CD34+ cell counts and rs11264422 genotype. Therefore, we suggest that the ethnic differences in subjects’ genetic background may be related to HSC mobilization. In conclusion, the relaxin—relaxin receptor axis may play an important role in HSC mobilization. We believe that the results of the current study could provide new insights for therapies that use relaxin and HSC populations, as well as a better understanding of HSC regulation and mobilization at the molecular level.

## Introduction

Hematopoietic stem cell (HSC) mobilization is a complex process that involves chemotactic factors, proteases, and adhesive molecules in bone marrow (BM) niches [1–3]. There is wide inter-individual variability in response to mobilization, and the outcome is hardly predictable despite several known demographic or clinical risk factors such as the following: age, sex, body mass index (BMI), ethnicity, diagnosis, and extent and duration of prior chemotherapy [4–8]. Inter-individual variation of HSC mobilization yield can be explained by a multifactorial model consisting of environmental and multiple genetic factors. Genetic contribution to mobilizing capacity is further supported by the fact that the second mobilization in the same donor typically yields similar results to those from the first mobilization [9,10].

Previous studies have reported genetic associations between single nucleotide polymorphisms (SNPs) and HSC mobilization yield [11–15]. Most of these SNPs are located in gene encoding molecules with known functional significance in the mobilization pathway, including C-X-C motif chemokine ligand 12 (*CXCL12*), vascular cell adhesion molecule 1 (*VCAM1*), CD44 (*CD44*), and colony stimulating factor 3 receptor (*CSF3R*) [11–15]. However, some of the results were not replicated in subsequent studies [11,16,17], and the responsible gene remains elusive.

Recent genome-wide association studies have shown that various hematologic traits of white blood cells (WBC), red blood cells, platelets, and CD34+ cells are highly heritable [18,19]. Previous studies have also indicated that each WBC subtype shares some associations which are probably attributable to shared process of differentiation and maintenance in BM and peripheral blood (PB) [18,20]. Therefore, we hypothesized that genetic factors associated with WBC count, neutrophil count, and circulating CD34+ cell count could also contribute to the regulation and migration of HSCs in BM niches and in PB.

The aim of this study was to identify genetic factors associated with HSC collection yield after mobilization in Korean population. We also attempted to determine whether our finding could be applied to other ethnic group of European ancestry.

## Methods

### Participants

A total of 148 Korean subjects, including 56 healthy donors for allogeneic HSC transplantation and 92 patients with hematologic disorders for autologous HSC transplantation, were prospectively recruited for this study. The European set was recruited to confirm the applicability of our findings, and consisted of 101 healthy donors of European ancestry from Germany. This study was approved by the institutional review board (IRB) of the Severance Hospital, Yonsei University College of Medicine (IRB No. 4-2013-0145). Written informed consent was obtained from all participants, in accordance with the Declaration of Helsinki.

### Mobilization and HSC collection

For healthy donors, standard mobilization protocol was used with G-CSF (filgrastim 10 μg/kg daily), and collection was initiated on the fifth day after G-CSF initiation. Mobilization for patients undergoing autologous HSC transplantation was performed using G-CSF only or chemotherapy followed by G-CSF. Apheresis started when the PB leukocyte count reached 3.0 x 10^9^/L after leukocyte nadir, in the case of combination with chemotherapy. Peak circulating CD34+ cell count (/μL), collected just before apheresis, was assessed using a Stem-Kit (Beckman Coulter, Miami, FL, USA) for the Korean set and with a BD Stem Cell Enumeration kit (BD Biosciences, San Jose, CA, USA) for the European set. The CD34+ cell content in the first apheresis product was enumerated in 122 participants in the Korean set, and two additional outcomes were evaluated: total CD34+ cell count per donor body weight (/kg) obtained from the first apheresis; and CD34+ cell count (/μL) from the first apheresis product.

### Selection of target polymorphisms in candidate genes

**Fig 1:**
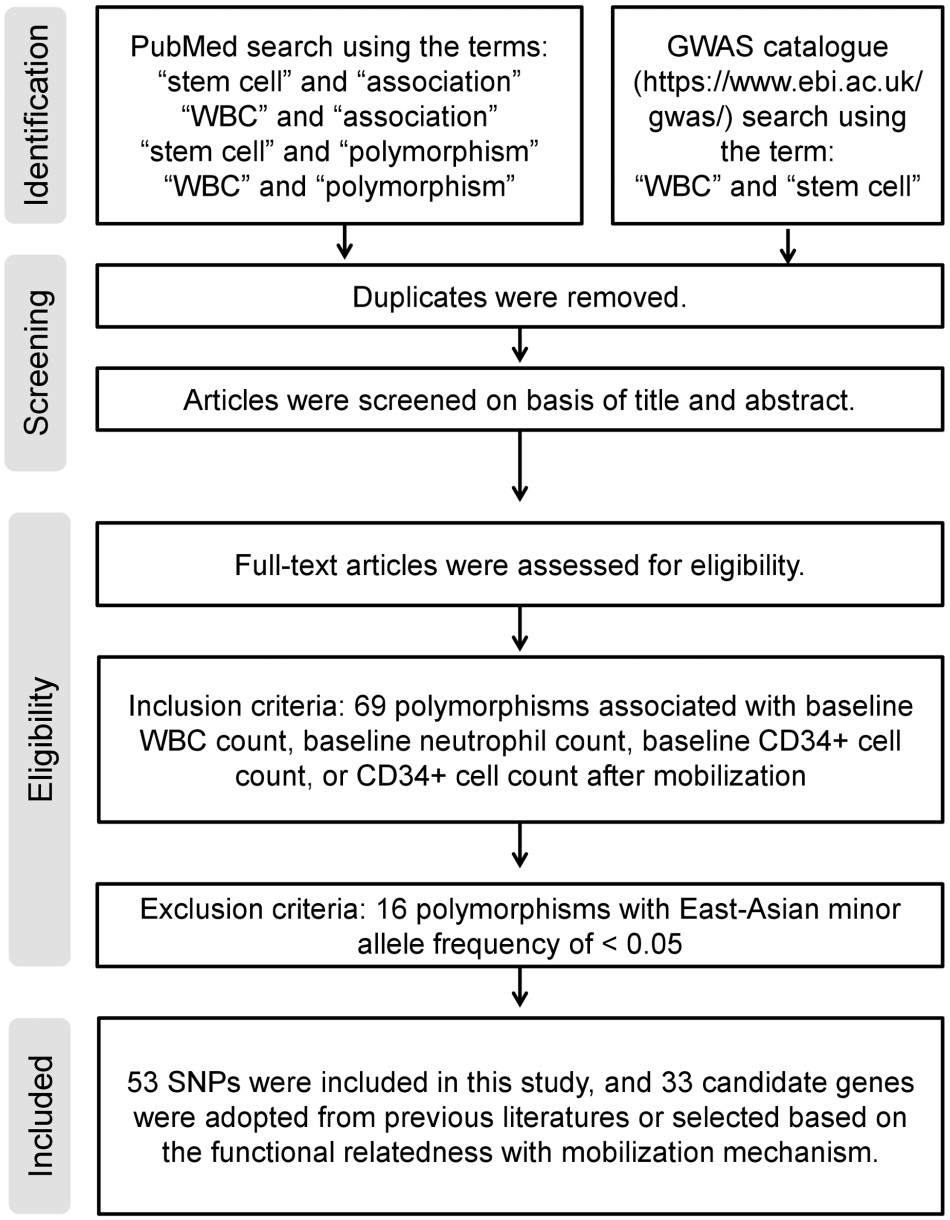
Flow diagram of target polymorphism selection. The diagram indicates inclusion and exclusion criteria for selection of target polymorphism.

To determine whether previously reported genetic associations with HSC yield might be applied to Koreans, we selected four common polymorphisms (rs1801157, rs1041163, rs13347, and rs3917924) in the following four genes: *CXCL12*, *VCAM1*, *CD44*, and *CSF3R* [11–17]. One polymorphism (rs2680880) in *CXCR4* was not included, as it was not found in East Asians (http://www.1000genomes.org/) [12]. To identify more candidate genes, we searched the literature for SNPs that are associated with WBC, neutrophil, or CD34+ cell counts [19–28] (Fig 1). Among the 64 additional SNPs, 15 with East Asian minor allele frequency of less than 0.05 were removed. Candidate genes were adopted from the literature or selected based on the functional relatedness to mobilization mechanism, such as cytokines, chemokines, proteases, and adhesion molecules (http://www.uniprot.org/) [2,3]. In total, 53 SNPs were selected for genotyping (Table 1).

**Table 1:** List of 53 polymorphisms in 33 genes included in this study.

rs ID	Chromosome	Location (GRCh38.p2)	Candidate gene	Distance to gene	Protein function
rs11121242	1p36.23	8846242	*RERE*	20 kb downstream	Control of cell survival
rs6577536	1p36.23	8850051	*RERE*	23 kb downstream	Control of cell survival
rs11590606	1p36.23	8857610	*RERE*	31 kb downstream	Control of cell survival
rs10864368	1p36.23	8858254	*RERE*	32 kb downstream	Control of cell survival
rs3917924	1p34.3	36480052	*CSF3R*	Intron2	Cell adhesion and chemotaxis
rs1041163	1p21.2	100718269	*VCAM1*	1 kb upstream	Cell adhesion and migration
rs6702883	1p21.1	104700458	intergenic		
rs4311917	1p13.3	107183121	*NTNG1*	Intron2	Controlling axon growth
rs345275	1p13.3	107951181	*VAV3*	Intron1	Regulation of cell adhesion
rs2365669	1p13.2	111820023	*KCND3*	Intron2	Subunit of potassium channel
rs7523839	1p13.2	115630459	*VANGL1*	11 kb upstream	Multicellular organism development
rs850610	1p13.1	116406090	*ATP1A1-AS1*	Exon 3	Non-coding RNA
rs10923929	1p12	119963373	*NOTCH2*	Intron11	Stem cell population maintenance
rs11240089	1q21.1	147588715	*BCL2*	Intron1	Cell migration
rs4657616	1q23.1	159001296	*ACKR1*	202 kb upstream	Chemokine receptor
rs2518564	1q23.1	159092646	*ACKR1*	111 kb upstream	Chemokine receptor
rs12075	1q23.1	159205564	*ACKR1*	Exon 2	Chemokine receptor
rs12740969	1q21.3	154514584	*TDRD10*	Intron4	Nucleotide binding
rs11264422	1q22	155938032	*RXFP4*	3 kb upstream	Relaxin-3 receptor
rs1962508	1q23.3	161975629	*DDR2*	655 kb upstream	Cell migration and remodeling of the extracellular matrix
rs2806424	1q23.3	162721669	*DDR2*	Intron4	Cell migration and remodeling of the extracellular matrix
rs6426893	1q23.3	165058105	intergenic		
rs919679	1q24.1	166287925	intergenic		
rs6734238	2q13	113083453	*IL1F10*	7 kb upstream	Cytokine
rs10932765	2q35	218234761	*ARPC2*	Intron5	Cytoskeleton constituent
rs16850408	4q13.3	74067090	*CXCL2*	29 kb upstream	Chemokine
rs546829	4q13.3	74090655	*CXCL2*	6 kb upstream	Chemokine
rs9131	4q13.3	74097332	*CXCL2*	Exon 4	Chemokine
rs7667376	4q13.3	74102173	*CXCL2*	2 kb downstream	Chemokine
rs1371799	4q13.3	74112120	*CXCL2*	12 kb downstream	Chemokine
rs7686861	4q13.3	74132767	*CXCL2*	33 kb downstream	Chemokine
rs2517524	6p21.33	31057936	*HCG22*	Intron3	Non-coding RNA
rs2853946	6p21.33	31279426	*HLA-B*	74 kb upstream	Regulation of immune response
rs2844503	6p21.33	31474954	*HLA-B*	117 kb downstream	Regulation of immune response
rs6936204	6p21.32	32249315	intergenic		
rs5020946	6p21.32	32482312	*BTNL2*	73 kb downstream	Regulation of T-cell proliferation
rs4895441	6q23.3	135105435	*MYB*	75 kb upstream	Control of proliferation and differentiation of hematopoietic progenitor cells
rs12660713	6q23.3	135196858	*MYB*	Intron9	Control of proliferation and differentiation of hematopoietic progenitor cells
rs976760	7p21.2	14234028	*DGKB*	Intron22	Intracellular signal transduction
rs445	7q21.2	92779056	*CDK6*	Intron2	Hematopoietic stem cell differentiation and cell adhesion
rs2163950	8q24.21	129585339	*CCDC26*	Intron 1	Non-coding RNA
rs579459	9q34.2	133278724	*ABO*	3 kb downstream	Blood group system
rs1801157	10q11.21	44372809	*CXCL12*	Exon 4	Chemokine
rs13347	11p13	35231725	*CD44*	Exon 18	Cell adhesion and migration
rs2183383	11p11.12	50279041	*PTPRJ*	2 Mb downstream	Regulation of cell adhesion
rs17609240	17q21.1	39954436	*GSDMA*	8 kb upstream	Pyroptosis mediator
rs3894194	17q21.1	39965740	*GSDMA*	Exon3	Pyroptosis mediator
rs3859192	17q21.1	39972395	*GSDMA*	Intron6	Pyroptosis mediator
rs4065321	17q21.1	39987295	*PSMD3*	Intron3	Regulatory subunit of the 26 proteasome
rs4794822	17q21.1	40000459	*CSF3*	14 kb upstream	Cytokine that controls granulocyte production
rs8078723	17q21.1	40010626	*CSF3*	4 kb upstream	Cytokine that controls granulocyte production
rs8065443	17q21.1	40052687	*MED24*	Intron3	Component of transcriptional coactivator complex
rs2072910	20p12.2	9384656	*PLCB4*	Intron13	Intracellular signal transduction

### SNP genotyping

Genomic DNA was extracted from PB leukocytes using the QIAamp DNA Blood Mini Kit (Qiagen, Venlo, The Netherlands). The primer sequences for polymerase chain reaction (PCR) amplification and sequencing were designed using Primer3 software [29]. PCR was performed on 100 ng of genomic DNA, and sequencing was carried out using the BrightDye Terminator Cycle Sequencing Kit (Nimagen, Nijmegen, The Netherlands) on ABI 3500 Genetic Analyzer (Applied Biosystems, Foster City, CA, USA). The results were compared with reference sequences using Sequencher 5.1 software (Gene Codes Corp., Ann Arbor, MI, USA). Quality of data was assessed using PHRED score for each base call [30]. The threshold for PHRED score was 20, based on the manufacturer’s instructions. In case of result with inadequate quality, sequencing was repeated and all genotype of tested locus were determined (no missing genotype data).

### Statistical analysis

Following the Kolmogorov—Smirnov normality test, natural log transformation was applied on continuous outcome variables with skewed distribution for analysis. The association between continuous variables (age and BMI) and mobilization outcomes (CD34+ cell count in PB, total CD34+ cells/kg, and CD34+ cells in a product) were analyzed using Pearson correlation. The association between categorical variables (sex, diagnosis, BM involvement of disease, chemotherapy regimen history, mobilization protocol, and SNP genotype), and mobilization outcomes were analyzed using an independent two-sample t-test (for two categories) and analysis of variance (for three categories). Three subgroups were established for the genotype of each polymorphism: homozygous for the major allele, heterozygous and homozygous for the minor allele. We also tested three genetic models (dominant, recessive, and additive) using biallelic marker coding. SNPs with a raw *P* < 0.05 in analysis with all three mobilization outcomes were included in multivariate linear regression analysis. Additional variables related to patient demographics or clinical history with *P* < 0.05 shown in univariate analysis were included in multivariate analysis. Finally, the following variables were included in multivariate analysis according to each mobilization outcome: 1) CD34+ cell count in PB: sex, diagnosis, chemotherapy regimen history, and rs11264422 (*RXFP4*) genotype; 2) total CD34+ cells/kg: sex and *RXFP4* genotype; and 3) CD34+ cell count in a product: sex, BMI, diagnosis, and *RXFP4* genotype. False discovery rate (FDR) controlling procedure was used to adjust for multiple testing according to the genetic model [31]. *P* values < 0.05 were considered significant, and *P* values < 0.2 after FDR adjustment were considered to have a tendency [32]. Statistical analysis was performed using SPSS Statistics version 23.0.0 (IBM Corp., Armonk, NY, USA). FDR adjusted *P* values were calculated using Microsoft Exel 2010 (Microsoft Corporation, Redmond, WA, USA).

## Results

### Patient characteristics

Patient characteristics are summarized in Table 2. The group consisted of individuals who were diagnosed with acute leukemia (n = 8), non-Hodgkin lymphoma (n = 50), multiple myeloma (n = 33), and sarcoma (n = 1). On the first day of apheresis, the median circulating CD34+ count was 44 cells/μL in the Korean set and 93 cells/μL in the European set (healthy donors only for the latter).

**Table 2:** Characteristics of the participants in this study.

	n (%)/median (interquartile ranges)	
Characteristics	Korean set	European set
No.	148	101
Age (yr)	46 (32–56)	32 (26–42)
Sex		
Female	63 (42.6)	26 (25.7)
Male	85 (57.4)	75 (74.3)
Body-mass index (kg/m^2^)	24.4 (21.5–26.1)	24.5 (22.4–28.0)
Diagnosis		
Healthy donor	56 (37.8)	101 (100)
Acute leukemia	8 (5.4)	-
Non-Hodgkin lymphoma	50 (33.8)	-
Multiple myeloma	33 (22.3)	-
Sarcoma	1 (0.7)	-
BM involvement of disease		
Present	51 (34.5)	-
Absent	97 (65.5)	-
Chemotherapy regimen history		
Multiple regimens (three or more)	9 (6.1)	-
One or two regimens	139 (93.9)	-
Mobilization		
Chemotherapy and G-CSF	80 (54.1)	-
G-CSF only	68 (45.9)	101 (100)
CD34+ cell count (/μL) in PB	44 (22–84)	93 (67–116)
First apheresis product*		
CD34+ cell count (/μL)	1,418 (591–2,330)	-
CD34+ cell count/kg donor (×10^6^)	3.54 (1.69–6.91)	-

PB, peripheral blood

^a^CD34+ cell count in an apheresis product was measured in 122 participants.

### Relaxin/insulin-like family peptide receptor 4

**Fig 2:**
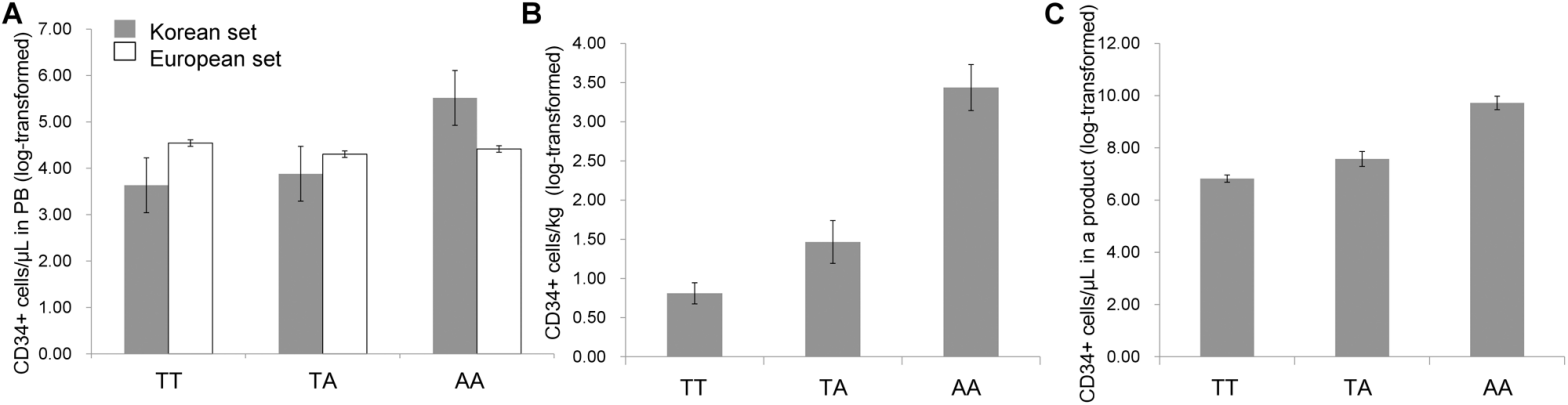
Correlations between rs11264422 genotype and continuous outcomes. There were significant associations between rs11264422 genotype and (A) circulating CD34+ cells/μL (raw *P* = 0.03), (B) total CD34+ cells/kg (raw *P* = 0.008), and (C) product CD34+ cells/μL (raw *P* = 0.003) in the Korean set (gray-colored bar). However, no statistically significant association was found between rs11264422 genotype and circulating CD34+ cells/μL in the European set (solid-lined bar). Mobilization outcomes were applied natural log transformation, due to the skewed distribution.

**Fig 3:**
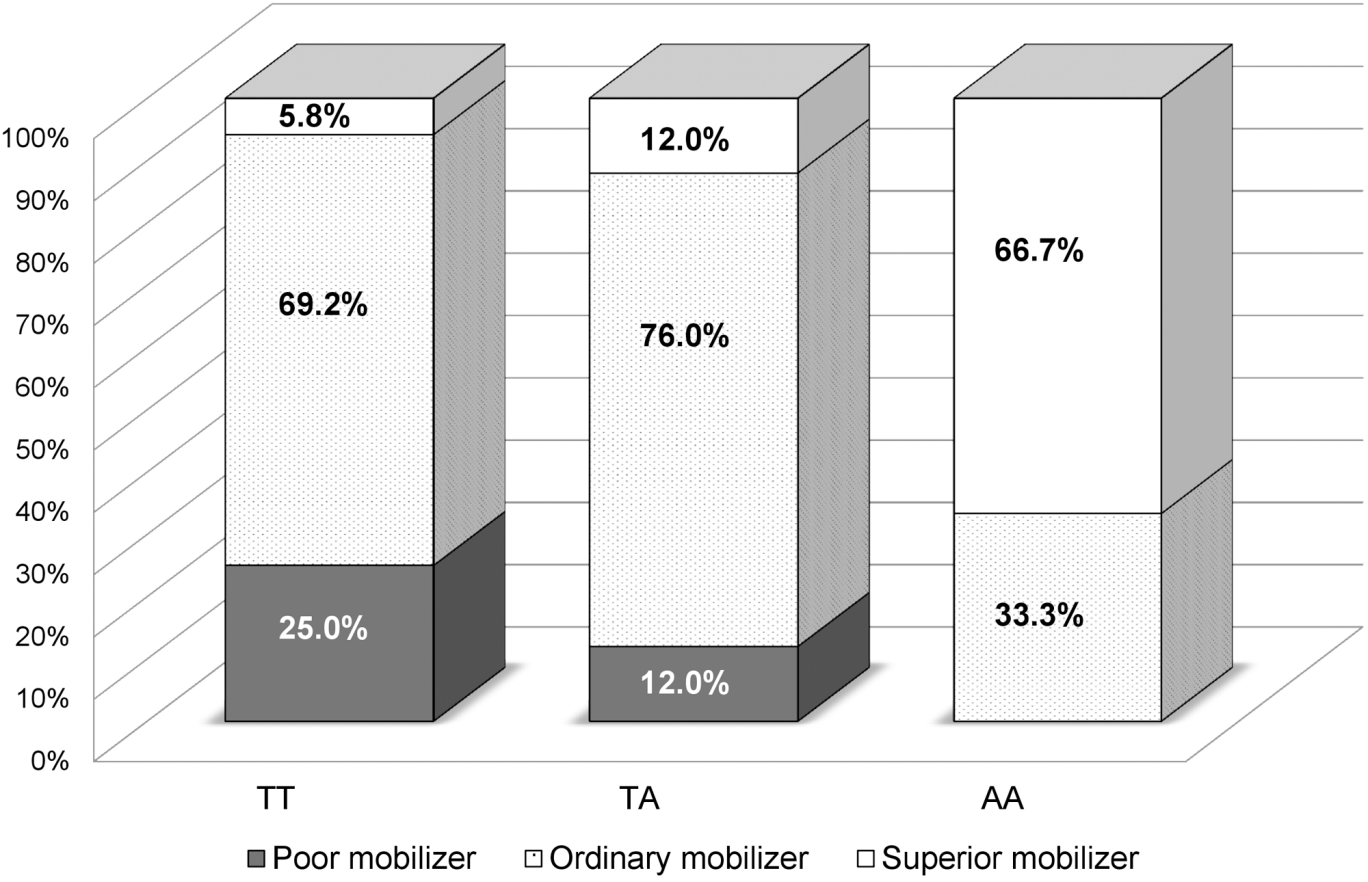
The rs11264422 genotype distribution of participants in the Korean set, classified by circulating CD34+ cell count. Superior mobilizers (> 200 cells/μL) had 66.7%, 12.0%, and 5.8% frequency rates in AA, TA, and TT genotypes, respectively. Poor mobilizers (< 20 cells/μL) had 25.0% and 12.0% frequency rates in TT and TA genotypes, respectively.

Of the 53 SNPs, only one polymorphism (rs11264422) made a significant difference in the three HSC mobilization outcomes of the Korean set (Table 3). The rs11264422 genotype, located 3 kb upstream of the relaxin/insulin-like family peptide receptor 4 (*RXFP4*) gene, was significantly associated with circulating CD34+ cells/μL (raw *P* = 0.03), total CD34+ cells/kg (raw *P* = 0.008), and product CD34+ cells/μL (raw *P* = 0.003) (Fig 2). Three patients (two with lymphoma and one with multiple myeloma) who were homozygous for a minor allele (AA genotype) showed remarkably higher mobilization outcomes compared to both the 25 patients who were heterozygous (TA genotype) and the 120 who were homozygous (TT genotype) for the major allele. Moreover, the presence of A allele (TA+AA genotypes) showed significant association with higher CD34+ cells/μL in a product (raw *P* = 0.02). Superior mobilizers (defined as > 200 circulating CD34+ cells/μL) had the highest frequency (66.7%) of the AA genotype, followed by TA (12.0%) and TT (5.8%) genotypes (Fig 3). In contrast, poor mobilizers (defined as < 20 circulating CD34+ cells/μL) had a higher frequency of the TT (25.0%) than TA (12.0%) genotype. However, for rs11264422 genotyping using the European set, the circulating CD34+ cell count did not differ between each genotype subgroup. SNP was at Hardy—Weinberg equilibrium in both Korean and European sets.

**Table 3:** Association of rs11264422 with mobilization outcomes^a^.

Genotype	Korean set												European set		
	CD34+ cells/μL in PB				CD34+ cells/kg (×10^6^)				CD34+ cells/μL in a product				CD34+ cells/μL in PB		
	n	Mean ± SD	Raw *P*	FDR *P*	n	Mean ± SD	Raw *P*	FDR *P*	n	Mean ± SD	Raw *P*	FDR *P*	n	Mean ± SD	*P*
TT	120	3.60 ± 1.28	0.03^b^*	0.808^b^	98	1.11 ± 1.33	0.008^b^*	0.424^b^	98	7.10 ± 1.39	0.003^b^*	0.159^b^***	11	4.54 ± 0.31	0.5^b^
TA	25	3.85 ± 1.17			21	1.47 ± 1.25			21	7.58 ± 1.31			41	4.30 ± 0.68	
AA	3	5.51 ± 0.51			3	3.44 ± 0.51			3	9.72 ± 0.45			49	4.41 ± 0.58	
TT+TA	145	3.55 ± 1.41	0.01^c^*	0.5^c^	119	1.20 ± 1.35	0.008^c^*	0.4^c^	119	7.25 ± 1.42	0.003^c^*	0.15^c^***	52	4.44 ± 0.54	0.3^c^
AA	3	5.51 ± 0.51			3	3.44 ± 0.51			3	9.72 ± 0.45			49	4.30 ± 0.63	
TT	120	3.60 ± 1.28	0.1^c^	0.728^c^	98	1.11 ± 1.33	0.05^c^**	0.795^c^	98	7.10 ± 1.39	0.02^c^*	0.711^c^	11	4.50 ±0.31	0.4^c^
TA+AA	28	4.02 ± 1.23			24	1.71 ± 1.35			24	7.84 ± 1.43			90	4.36 ± 0.63	
TT+AA	123	3.65 ± 1.33	0.5^c^	0.995^c^	101	1.23 ± 1.37	0.4^c^	0.938^c^	101	7.22 ± 1.44	0.3^c^	0.88^c^	60	4.41 ± 0.58	0.5^c^
TA	25	3.84 ± 1.17			21	1.47 ± 1.25			21	7.57 ± 1.31			41	4.35 ± 0.63	

PB, peripheral blood; SD, standard deviation; FDR, adjusted *P* value using false discovery rate controlling procedure

^a^Natural log transformation was applied to mobilization outcomes due to skewed distribution.

^b^Analysis of variance

Independent two-sample t-test

*P< 0.05

**P = 0.05

***P< 0.2 after FDR adjustment

### Univariate and multivariate analyses of host factors and mobilization outcomes

In univariate analysis, the circulating CD34+ cell count after mobilization was associated with sex, diagnosis, history of multiple chemotherapy regimens, and *RXFP4* genotype in the Korean population (Table 4). In the European set, only a low BMI showed significant correlation with a low circulating CD34+ cell count (*P* < 0.001). In the Korean set, the total CD34+ cell count/kg was associated with sex and *RXFP4* genotype, while the CD34+ cell count in a product was associated with sex, BMI, diagnosis, and *RXFP4* genotype.

**Table 4:** Factors associated with mobilization outcomes in the univariate analysis^a^.

Variables	Korean set						European set	
	CD34+ cells/μL in PB		CD34+ cells/kg (×10^6^)		CD34+ cells/μL in a product		CD34+ cells/μL in PB	
	r, mean ± SD	*P* value	r, mean ± SD	*P* value	r, mean ± SD	*P* value	r, mean ± SD	*P* value
Age (yr)	-0.064	0.4^b^	-0.014	0.9^b^	0.096	0.3^b^	0.007	0.9^b^
Sex		0.001^c^*		0.002^c^*		<0.001^c^*		0.06^c^
Male	3.99 ± 1.20		1.87 ± 1.01		7.70 ± 1.42		4.47 ± 0.51	
Female	3.34 ± 1.19		1.34 ± 0.77		6.65 ± 1.19		4.15 ± 0.78	
Body-mass index (kg/m^2^)	0.155	0.06^b^	0.112	0.2^b^	0.204	0.02^b^*	0.343	<0.001^b^*
Diagnosis		<0.001^d^*		0.08^d^		0.01^d^*	-	
Healthy donor	3.88 ± 0.57		1.74 ± 0.39		6.81 ± 0.58			
Acute leukemia	2.07 ± 1.04		0.81 ± 0.55		6.16 ± 1.27			
Non-Hodgkin lymphoma/sarcoma^e^	3.73 ± 1.68		1.69 ± 1.22		7.61 ± 1.05			
Multiple myeloma	3.87 ± 0.96		1.67 ± 0.95		7.61 ± 1.05			
BM involvement of disease		0.2^c^		0.4^c^		0.4^c^	-	
Absent	3.80 ± 1.17		1.69 ± 0.94		7.16 ± 1.43			
Present	3.55 ± 1.35		1.56 ± 0.96		7.37 ± 1.40			
Chemotherapy regimen history		0.04^c^*		0.09^c^		0.3^c^	-	
One or two regimens	3.77 ± 1.22		1.68 ± 0.95		7.29 ± 1.41			
Multiple regimens (three or more)	2.89 ± 1.27		1.14 ± 0.85		6.72 ± 1.52			
Mobilization		0.8^c^		0.9^c^		0.7^c^	-	
G-CSF only	3.69 ± 1.28		1.64 ± 0.97		7.30 ± 1.45			
Chemotherapy and G-CSF	3.74 ± 1.20		1.64 ± 0.93		7.19 ± 1.40			
*RXFP4* genotype		0.03^d^*		0.008^d^*		0.003^d^*		0.5^d^
TT	3.60 ± 1.28		1.11 ± 1.33		7.10 ± 1.39		4.54 ± 0.31	
TA	3.85 ± 1.17		1.47 ± 1.25		7.58 ± 1.31		4.30 ± 0.68	
AA	5.51 ± 0.51		3.44 ± 0.51		9.72 ± 0.45		4.41 ± 0.58	

^a^Natural log transformation was applied to mobilization outcomes due to skewed distribution. Data were represented as correlation coefficient (r) or mean ± standard deviation.

^b^Pearson correlation test

^c^Independent two-sample t-test

^d^Analysis of variance

^e^50 patients with non-Hodgkin lymphoma and one with sarcoma were included.

*P < 0.05

Multivariate linear regression analysis revealed that female sex, diagnosis of acute leukemia, history of multiple chemotherapy regimens, and *RXFP4* genotype (TT and TA) remained independently associated with lower circulating CD34+ cell count after mobilization in the Korean set (Table 5). Female sex and *RXFP4* genotype (TT and TA) showed consistent significance when analyzed with other outcome variables, i.e., total CD34+ cell count/kg and CD34+ cell count in a product.

**Table 5:** Factors associated with log-transformed mobilization outcomes in the multivariate linear regression analysis in the Korean set.

Variables	CD34+ cells/μL in PB		CD34+ cells/kg (×10^6^)		CD34+ cells/μL in a product	
	β (95% CI)	*P* value	β (95% CI)	*P* value	β (95% CI)	*P* value
Sex						
Male	Reference		Reference		Reference	
Female	-0.660 (-1.028, -0.292)	0.001*	-0.630 (-1.018, -0.242)	0.002*	-0.590 (-0.987, -0.193)	0.004*
Body-mass index (kg/m^2^)					0.025 (-0.031, 0.081)	0.4
Diagnosis						
Healthy donor	Reference				Reference	
Acute leukemia	-1.722 (-2.545, -0.898)	<0.001*			-1.737 (-2.574, -0.901)	<0.001*
Non-Hodgkin lymphoma/sarcoma^a^	-0.259 (-0.701, 0.183)	0.2			-0.368 (-0.803, 0.068)	0.09
Multiple myeloma	-0.057 (-0.551, 0.437)	0.8			-0.150 (-0.653, 0.353)	0.6
Chemotherapy regimen history						
Multiple regimens (three or more)	Reference					
One or two regimens	0.877 (0.100, 1.654)	0.03*				
*RXFP4* genotype						
TT	Reference		Reference		Reference	
TA	0.091 (-0.405, 0.588)	0.7	0.166 (-0.347, 0.678)	0.5	0.122 (-0.382, 0.626)	0.6
AA	1.735 (0.446, 3.024)	0.009*	1.809 (0.452, 3.166)	0.009*	1.830 (0.526, 3.135)	0.006*

PB, peripheral blood; CI, confidence interval

^a^50 patients with non-Hodgkin lymphoma and one with sarcoma were included.

*P < 0.05

## Discussion

In this study, we found that rs11264422 genotype, located in the promoter flanking region of *RXFP4*, has a significant effect on HSC mobilization. The *RXFP4* gene encodes relaxin-3 receptor 2, which is a receptor for relaxin-3 and is expressed in various tissues including BM [33]. Relaxin-3 is a member of the insulin/relaxin superfamily of peptide hormones [34]. Segal *et al*. revealed that the relaxin hormone mobilizes BM-derived CD34+ endothelial progenitor cells into circulation, and their effect is mediated by the relaxin receptor [35]. The role of relaxin and its receptor-mediated pathway in HSC mobilization, as well as their association with the inter-individual variation of mobilization yield, can be hypothesized based on such observation.

The FDR-adjusted *P*-values for rs11264422 were above the significance threshold (*P* = 0.05). However, we considered *P* < 0.2 after FDR adjustment as having a tendency for association. Given that the sample size was inadequate compared with the number of genes, we sought to find a possible exploratory factor. We determined three different mobilization outcomes and found consistent genes in all three. We then decided that the *P*-value of rs11264422 showed a meaningful trend, and wanted to suggest a further study. Therefore, we would like to conduct a confirmatory study using a larger number of patients.

The rs11264422 polymorphism has been associated with lower WBC counts in individuals of African, but not European, ancestry [28]. In our study, rs11264422 genotype was associated with HSC yield in Koreans but not in Europeans. Interestingly, the frequency of AA homozygote genotype is low in East Asians (1‒4% in Japanese and Chinese) and Africans (0.2%), but distinctly higher in Europeans (43%). Moreover, in a previous randomized controlled trial in Japan, a higher baseline WBC count was associated with a lower incidence of poor mobilization [36]. Therefore, we infer that the mechanism involved in HSC mobilization differs by ethnic groups, and rs11264422 genotype is associated with the HSC mobilization yield as well as the baseline WBC count in certain populations. Moreover, associations between the four polymorphisms in *CXCL12*, *VCAM1*, *CD44*, and *CSF3R* and mobilization outcome were not replicated in our study. Previous studies have already noted discrepancies in genetic associations, which were likely attributed to differences in ethnicity, diagnosis, number of study participants, and definition of outcome [13–17]. In particular, most of the previous studies had targeted those of European ancestry, whereas our study is the first to target the East Asian population. Therefore, our results suggest that there are significant differences in molecular mechanisms underlying HSC mobilization between different ethnic groups. Our preliminary data warrant further validation with larger cohorts of various population subgroups.

The therapeutic effect of circulating CD34+ cells has been demonstrated in hematologic disorders and cardiovascular diseases [37,38]. In this context, the promotion of vasculogenesis is thought to be a mechanism for efficacy of CD34+ progenitor cells [19]. Notably, serelaxin, which is a recombinant human relaxin-2, has demonstrated significant treatment effects on acute heart failure in a recent clinical trial [39]. The potential mechanisms behind beneficial effects of serelaxin in acute heart failure include vasodilation, tissue healing from stimulation of angiogenesis and stem cell survival, and remodeling of the extracellular matrix [40]. Furthermore, a recent experimental study demonstrated that relaxin improves wound healing in diabetic mice [41]. In that study, the wound-healing effect of relaxin was disturbed by antibodies against vascular endothelial growth factor, CXCR4, and CXCR12 [41]. Our data support previous assumptions about the effects of relaxin on vasculogenic capacity and stem cell/progenitor cell regulation, and suggest a broader applicability of relaxin to other vascular disorders such as diabetes mellitus. In addition, our data also suggest that relaxin is a novel agent for the management of poor mobilizers.

Among host risk factors, female sex, history of multiple chemotherapy regimens, and diagnosis of acute leukemia remained independently associated with low circulating CD34+ cell counts in Koreans. Female sex [4,42], prior treatment history [1], and diagnosis of acute leukemia [43] have all been known to be independent risk factors for poor mobilization. The mechanism behind association of sex and better mobilization potential can be explained by the stem cell regulation effect of sex steroids [44]. The contribution of an underlying hematologic disease on HSC mobilization can be explained by disease-related reduction of HSC reservoir, or chemotherapy-induced toxic effects on BM [43]. In the European set, only BMI correlated with circulating CD34+ cell counts. The mechanism behind association between higher BMI and better mobilization potential has been attributed to the effect of adipose tissue-containing HSCs, or a simple dose effect of G-CSF [8].

To the best of our knowledge, this is the first study to indicate an association between relaxin receptor polymorphism and HSC yield after mobilization. A potential limitation of our study is that the discovered locus is located in the regulatory region of *RXFP4*, and not in the protein-coding region. Further investigation regarding the functional effect of relaxin-3, as well as its receptor axis on the mobilization process, are required.

In conclusion, we found a novel association between relaxin receptor polymorphism and HSC yield after mobilization in ethnic Koreans. Our findings suggest an important functional role of relaxin axis during response of BM HSCs to the mobilizing agent. Results of our study give valuable insight to a potential therapeutic target—the relaxin—relaxin receptor axis—for the management of poor mobilizers, and for the treatment of various vascular diseases.

## Supporting information

S1 File Table A. Association of 53 polymorphisms with mobilization outcomes.
(XLSX)
