# Correction: A novel association between relaxin receptor polymorphism and hematopoietic stem cell yield after mobilization

**DOI:** 10.1371/journal.pone.0225278

**Published:** 2019-11-07

**Authors:** Saeam Shin, Juwon Kim, Soo-Zin Kim-Wanner, Halvard Bönig, Sung Ran Cho, Sinyoung Kim, Jong Rak Choi, Kyung-A Lee

The Data Availability statement is incorrect. The raw genotyping data underlying the study are not provided in the published paper and its Supporting Information files. The authors have provided the data as Supporting Information file S1 Data.

## Supporting information

S1 Data Supplementary dataset. Raw genotyping data.
(XLSX)
